# Research Progress of Bile Acids in Cancer

**DOI:** 10.3389/fonc.2021.778258

**Published:** 2022-01-20

**Authors:** Junhao Fu, Min Yu, Wenxia Xu, Shian Yu

**Affiliations:** ^1^ Central Laboratory, Affiliated Jinhua Hospital, Zhejiang University School of Medicine, Jinhua, China; ^2^ Department of Hepatobiliary and Pancreatic Surgery, Affiliated Jinhua Hospital, Zhejiang University School of Medicine, Jinhua, China

**Keywords:** bile acids, cancer, angiogenesis, proliferation and death, metastasis, inflammation and immunity

## Abstract

Bile acids (BAs) were originally known as detergents to facilitate the digestion and absorption of lipids. And our current knowledge of BAs has been extended to potential carcinogenic or cancer suppressor factors due to constant research. In fact, BAs were regarded as a tumor promoters as early as the 1940s. Differential bile acid signals emitted by various bile acid profiles can produce distinct pathophysiological traits, thereby participating in the occurrence and development of tumors. Nevertheless, in recent years, more and more studies have noticed the value of BAs as therapeutic targets. And several studies have applied BAs as a therapeutic agent for various diseases including cancer. Based on the above evidence, we acknowledge that the role of BAs in cancer has yet to be exploited, although considerable efforts have been made to probe the functions of BAs. In this review, we describe the characteristics of BAs as a double-edged sword in cancer, hoping to provide references for future cancer treatments.

## Introduction

Cancer occurrence is a multifactorial process and has emerged as the second leading cause of death in the world (1). As per the GLOBOCAN 2020 cancer data provided by the International Agency for Research on Cancer, there were an estimated 10.0 million cancer-responsible deaths in 2020, while an estimated 19.3 million new cancer cases emerged (2). As one of the most challenging diseases, cancer seriously threatens people’s quality of life, which imposes alarming situation of cancer. When normal cells transform into a tumor status, they will acquire a series of hallmark capabilities, that is, the characteristics of tumor cells, including immortal proliferation, resistance to cell death, and induction of angiogenesis, etc (3). A comprehensive understanding of these concepts will increasingly influence the emergence of new options for cancer treatment.

Bile acids (BAs), synthesized from cholesterol in the liver, are not only emulsifiers that promote lipid digestion and absorption, but also serve as signal molecules to perform different biological functions. The role of BAs in cancer has always attracted much attention, but its capabilities have not been finalized although extensive research has been conducted. In the 1940s, bile acids were initially considered to be tumor promoters due to the tumorigenic effects of deoxycholic acid (secondary bile acid) (4). Beyond tumorigenicity, the therapeutic potential of BAs has gradually been tapped in recent years (5–7). The protective or toxic effects of BAs are affected by many factors, including the species and concentration of BAs, and cell types, etc (8–12). The contradictory role played by BAs endow them with great heterogeneity, which in turn leads to the complexity of diagnosis and treatment. This review discusses the synthesis and circulation of BAs, with a focus on the role of BAs in cancer, intending to provide potential options for cancer treatment.

## Biosynthesis of Primary Bile Acids

**Figure 1:**
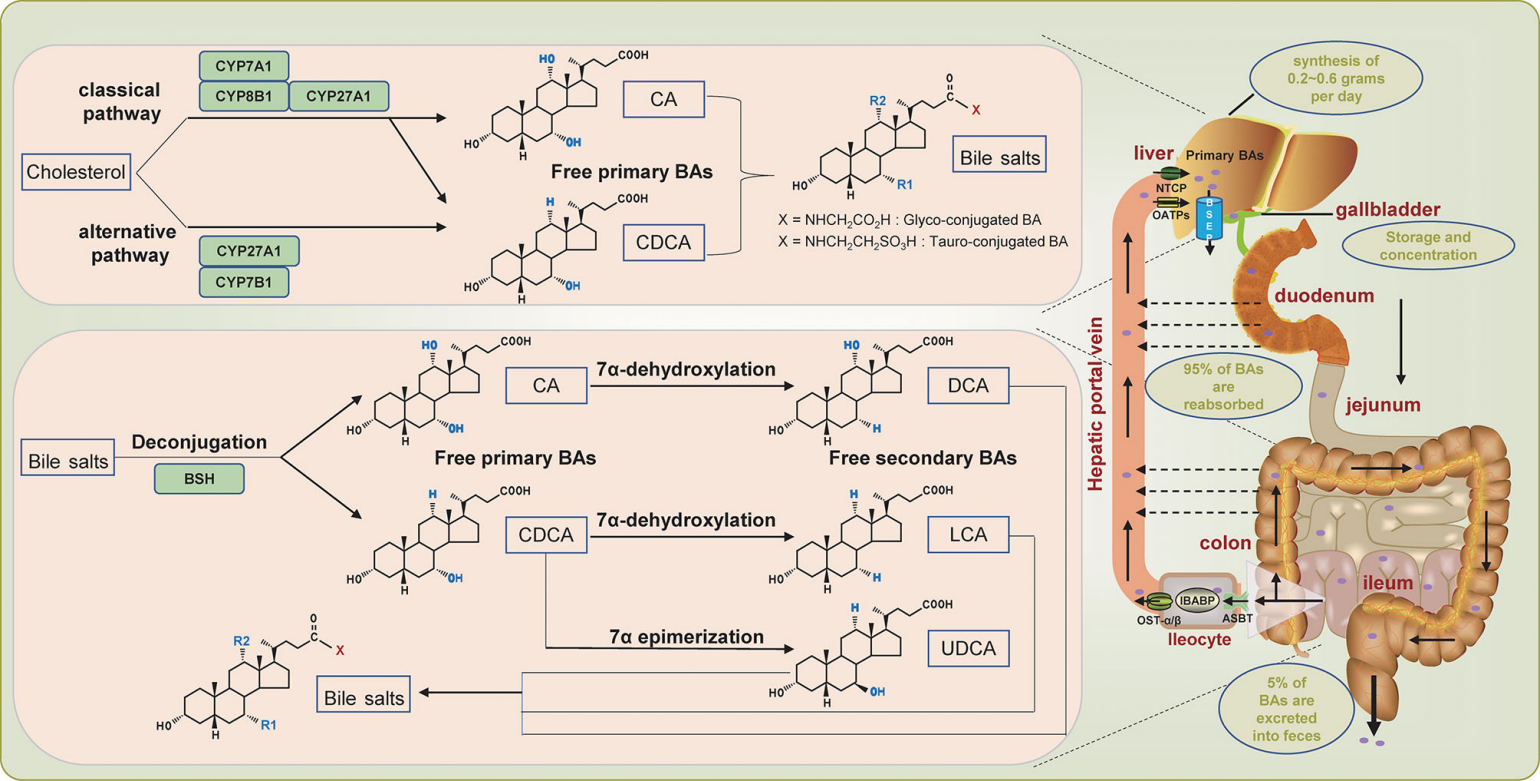
Overview of enterohepatic circulation of bile acids. The dashed arrow indicates passive absorption.

BAs are amphiphilic molecules produced by a series of enzymatic reactions with cholesterol as a substrate and are the primary metabolite of cholesterol in the body (13). The human bile acid pool is composed of primary bile acids and secondary bile acids. The synthesis of primary bile acids involves two different pathways, defined as the classical pathway and the alternative pathway, which are also called the neutral pathway and the acid pathway, respectively (14, 15) (**Figure 1**). Under normal circumstances, the classical pathway, responsible for about 90% of bile acid production, is considered the main pathway for bile acid synthesis. Cholesterol 7α-hydroxylase (CYP7A1) located in the endoplasmic reticulum initiates the classical pathway and is the rate-limiting enzyme of this pathway. Then go through the sterol 12α-hydroxylase (CYP8B1) branch and the sterol 27-hydroxylase (CYP27A1) branch to form the primary bile acids cholic acid (CA) and chenodeoxycholic acid (CDCA), respectively (**Figure 1**). As for the alternative pathway, it is initiated by CYP27A1, which converts cholesterol into 27-hydroxycholesterol through a hydroxylation reaction. 27-hydroxycholesterol is subsequently converted into CDCA instead of CA with the participation of oxysterol 7α hydroxylase (CYP7B1) (**Figure 1**). The alternative pathway is the secondary pathway of bile acid synthesis, accounting for about 10% of bile acid production, which is generally considered to be activated under pathological states (16–19). After the synthesis of primary bile acids, taurine or glycine is conjugated to it in a ratio of 1:3 through covalent modification (as known as bile salts), which improves its solubility while reducing toxicity (7).

## Transport of Bile Acids

To maintain the versatility of the bile acid pool by enhancing the utilization of bile acids, the body has designed a complex transport system involving the liver, intestines, and kidneys to extensively circulate the limited bile acids, which is defined as the well-known enterohepatic circulation of BAs. The conjugated primary bile acids are first secreted into the bile duct through the bile salt export pump (BSEP), and then stored and concentrated in the gallbladder (20) (**Figure 1**). After meals, cholecystokinin is secreted and stimulates the contraction of the gallbladder to promote the excretion of bile acids into the intestinal lumen, aiming to assist the digestion and absorption of food (21). In the intestine, the conjugated primary bile acids are catalyzed by bile salt hydrolase (BSH) secreted by gut bacteria, which deconjugates and reconverts the conjugated primary bile acids into free primary bile acids CA and CDCA(**Figure 1**). The multi-step 7α-dehydroxylation pathway continues to perform dehydroxylation reactions on CA and CDCA to form secondary bile acids: CA is converted into deoxycholic acid (DCA), and CDCA is converted into lithocholic acid (LCA) and ursodeoxycholic acid (UDCA) (22). The secondary bile acids can also be conjugated to taurine or glycine to form conjugated bile acids. Of the secreted bile acids, an estimated 95% are efficiently reabsorbed in the ileum through active transport, while only an estimated 5% are excreted into feces (7, 23) (**Figure 1**). Specifically, the distal ileum first actively uptakes bile acids through the apical sodium-dependent bile acid transporter (ASBT). The bile acids absorbed into the intestinal cells are transferred to the basolateral membrane mediated by ileal bile acid binding protein (IBABP), where the bile acids are discharged to the portal blood through the organic solute transporter-α/β (OST-α/β), and subsequently transported to the liver. In the liver, hepatocytes re-uptake bile acids through sodium taurocholate co-transport polypeptide (NTCP) and organic anion transporting polypeptides (OATPs) (24–27) (**Figure 1**). The human body goes through this cycle 6 to 8 times per day, which exaggerates the production of bile acids synthesized *de novo* (about 0.2~0.6 grams per day), thereby maintaining a functional bile acid pool (about 3 grams) (15, 17, 27).

## Bile Acids in Cancer Angiogenesis

Angiogenesis is the process of establishing new blood vessels based on existing blood vessels. Tumor angiogenesis is capable of providing oxygen and nutritional support for tumor cells, which is a vital factor for tumor growth and proliferation (28). The normal vasculature, that is, when the intravascular pro-angiogenic factors and anti-angiogenic factors are in balance, is basically static. In contrast, during tumorigenesis, the vascular homeostasis tilts towards pro-angiogenic factors, while anti-angiogenic factors are inhibited, resulting in the continuous sprouting of novel blood vessels, which is also known as the activation of the “angiogenic switch” (3, 29). Tumor angiogenesis involves a highly complex signal network. Therefore, traditionally stand-alone anti-angiogenic drugs, such as VEGF/VEGFR targeted inhibitors, cannot achieve satisfactory therapeutic effects (30, 31). In this regard, the discovery of new potential targets or the combination of anti-angiogenic drugs with other therapies including chemotherapy or immunotherapy seems to achieve the desired goals (32–34).

In addition to the extensively researched pathways such as VEGF/VEGFR, PDGF/PDGFR, and FGF/FGFR, bile acids also play a promising role in tumor angiogenesis to some extent, even with limited information (**Table 1**). In the late 19th century, some scholars have discovered the anti-angiogenic activity of UDCA and its derivatives, suggesting the potential of BAs for the treatment of diseases that are accompanied by uncontrolled angiogenesis, including tumors (39). In contrast, a subsequent study on liver cancer found that BAs levels were positively correlated with the expression levels of VEGFR-2 and CD34, that is, BAs promoted tumor angiogenesis in liver cancer (35). Coincidentally, another study showed that CDCA promotes esophageal cancer angiogenesis and tumor growth *via* the COX-2 pathway (36). It was also found that TUDCA can promote vascular repair and stimulate angiogenesis by recruiting vasculogenic progenitor cells (40). In addition to directly participating in angiogenesis, BAs and their derivatives can also be used as a mediator to complex with heparin (with anti-angiogenesis and anti-cancer activity) to increase the absorption of heparin in the intestines, thereby indirectly exerting anti-angiogenesis effects (38, 41–43). Reports on the role of BAs in cancer angiogenesis are limited, and people prefer to develop the mediator capacity of BAs based on their amphipathic nature to improve the absorption of anti-angiogenic agents. In fact, BAs frequently perform the role of promoting cancer angiogenesis due to their cytotoxicity by stimulating the secretion of pro-angiogenic factors and recruiting angiogenic precursor cells. Research gaps on the direct anti-angiogenic effects of BAs allow us to further explore.

**Table 1:** The impact of bile acid on cancer angiogenesis.

Bile acid	Target	Cancer	Model(s)	Effect
Total bile acids (TBAs)	Endothelial progenitor cell (EPC)	Hepatocellular carcinoma	Humans	Promote (35)
CDCA	COX-2	Esophageal cancer	Cells and mice	Promote (36)
LCA	Erk1/2-STAT3-IL-8	Colorectal cancer	Cells	Promote (37)
DCA-heparin conjugate	Basic fibroblast growth factor (bFGF)	Murine squamous cell carcinoma	Cells and mice	Suppress (38)

## Bile Acids in Cancer Cell Proliferation and Death

Long-term sustained proliferation can be ranked as the most basic attribute of cancer cells. Normal cells strictly control the generation and release of growth signals, while cancer cells deregulate these signal networks through a variety of ways, such as producing growth factor ligands themselves or stimulating normal cells to feed back growth factors (3, 44). Sustaining proliferation signal of cancer cells is habitually accompanied by the emergence of death resistance. Similarly, normal cells will evolve multiple alternative mechanisms to circumvent death during the process of malignant transformation, including destroying vital sensors in cell death circuits, up-/down-regulating anti-/pro-death regulators, and blocking extrinsic ligand-induced death pathways, etc (3).

The degree of hydrophobicity of bile acid (the order of hydrophobicity is: LCA> DCA> CDCA> UDCA), which is highly correlated with the number and position of hydroxyl groups attached to it, is a fundamental determinant of its biological activity (13). After diligent research, the previous concept that hydrophobic bile acids act as carcinogens is no longer completely convincing (**Table 2**). For the most hydrophobic LCA, the most impressive is the use of its cytotoxicity to exert anti-tumor effects. For instance, Miko et al. found that LCA can inhibit the proliferation of breast cancer cells by activating the TGR5 receptor (45). Following, they confirmed that LCA suppresses the proliferation of breast cancer cells by causing oxidative stress (46). Coincidentally, Luu et al. also declared that LCA exerts anti-proliferation and pro-apoptosis effects in breast cancer cells by inducing the expression of TGR5 (47). In addition, LCA has also shown surprising anti-tumor growth activity in a variety of other tumors, including human prostate cancer, human nephroblastoma, neuroblastoma and liver cancer, etc (48–51). Similar to LCA, the primary effect of DCA, which is ranked second in hydrophobicity, is also an anti-tumor effect. Lin et al. found that DCA can limit the proliferation of gallbladder cancer cells, and further, DCA treatment can significantly inhibit the growth of gallbladder cancer xenografted tumor in nude mice (12). Yang et al. pointed out that on the one hand, DCA can inhibit the proliferation of gastric carcinoma cells by arresting the cell cycle in the G0/G1 phase, and on the other hand, it can induce apoptosis of gastric carcinoma cells *via* a p53-mediated pathway (52). Jang et al. stated that DCA accelerates the apoptosis of NTCP-positive liver cancer cells by inducing endoplasmic reticulum stress, especially under hypoxic conditions (53). Paradoxically, Qiao et al. showed that DCA can activate ERK in intestinal cancer cells and that elevated ERK activity can in turn inhibit DCA-induced apoptosis (54). Pai et al. found that low concentrations of DCA can activate the β-catenin pathway and stimulate the expression of urokinase plasminogen activator (uPA), urokinase plasminogen activator receptor (uPAR), and cyclin D1, further promoting the proliferation of colon cancer cells (55). Milovic et al. also confirmed that low-dose DCA can stimulate colon cancer cell proliferation (56). Zhu et al. observed that DCA accelerates the proliferation of colorectal cancer cells by activating stromal COX-2 signals (57). Beyond colorectal cancer, Chen et al. also reported that DCA can induce the transformation of esophageal adenocarcinoma stem cells and improve their anti-apoptotic ability (58). The hydrophobic third-place CDCA also exhibits similar contradictory effects to DCA. For example, Powell et al. showed that CDCA can swiftly induce apoptosis of colon cancer cells (59). Ignacio et al. also proved that CDCA induces apoptosis of human colon adenocarcinoma cells through oxidative stress, and this effect of CDCA is mightier than that of DCA (60). Instead, Casabri et al. suggested that low-dose CDCA induces the expression of cyclin D1 by activating the TGR5-dependent CREB signal, thereby promoting the proliferation of endometrial cancer cells (61). Interestingly, Dai et al. reached the opposite conclusion in cholangiocarcinoma, that CDCA suppressed the proliferation of cholangiocarcinoma cells, and the same phenomenon was also observed in mice (62). Speaking of UDCA, which has the weakest hydrophobicity, the most attractive thing is its anti-cancer properties. Yu et al. discovered that UDCA prompted human melanoma cell apoptosis through mitochondrial-related pathways triggered by ROS (63). Lim et al. observed that UDCA induces the death of drug-resistant gastric carcinoma cells through the autophagy pathway (64). Yao et al. verified that UDCA inhibits the progression of glioblastoma through cell cycle arrest and apoptosis mediated by endoplasmic reticulum stress (65). The clues of UDCA’s anti-proliferation and pro-apoptosis effects have also been located in a variety of cancers, including liver cancer, prostate cancer, and colon cancer (66–68). In addition, Dai et al. demonstrated that conjugated bile acids (CBAs) promote the growth of cholangiocarcinoma from the cellular and mice levels, while free bile acids inhibit the growth of cholangiocarcinoma (69). This part of the research is the most intensive and typically focuses on cell models. Among them, we noticed the contradictory role of DCA and CDCA in cancer cell proliferation and death. We found that the effects of DCA and CDCA on promoting cell proliferation or resisting cell death generally occur at low doses (within 50 μM), while anti-cell proliferation or pro-cell death regularly occurs at high doses (beyond 100 μM), despite the specific molecular mechanism need to be further covered. In general, the role of BAs in cancer cell proliferation and death is closely related to their type and concentration.

**Table 2:** The impact of bile acid on cancer cell proliferation and death.

Bile acid	Target	Cancer	Model(s)	Effect
LCA (or LCA-conjugate)	TGR5	Breast cancer	Cells, mice, and humans	Suppress proliferation (45)
	Oxidative stress	Breast cancer	Cells, mice, and humans	Suppress proliferation (46)
	TGR5	Breast cancer	Cells	Suppress proliferation and promote death (47)
	ER stress/autophagy/mitochondrial	Prostate cancer	Cells	Promote death (48)
	Caspase 3/7	Nephroblastoma	Cells	Promote death (49)
	Intrinsic mitochondrial apoptotic cell death pathway/extrinsic death receptor pathway of apoptosis	Neuroblastoma	Cells	Promote death (50)
	Reactive oxygen species (ROS)	Liver cancer	Cells	Promote death (51)
DCA	miR-92b-3p-PTEN-PI3K/AKT	Gallbladder cancer	Cells, mice, and humans	Suppress proliferation (12)
	Cell cycle progression/intrinsic mitochondrial apoptotic cell death pathway	Gastric carcinoma	Cells	Suppress proliferation and promote death (52)
	ER stress	Hepatocellular carcinoma	Cells	Promote death (53)
	ERK	Colorectal cancer	Cells	Promote/suppress death (54)
	β-catenin–cyclin D1 and –uPAR	Colorectal cancer	Cells	Promote proliferation (55)
	/	Colorectal cancer	Cells	Promote proliferation (56)
	COX-2	Colorectal cancer	Cells	Promote proliferation (57)
	IL-6/STAT3-KFL4, OCT4	Esophageal Adenocarcinoma	Cells	Suppress death (58)
CDCA	/	Colorectal cancer	Cells	Promote death (59)
	Oxidative stress	Colon adenocarcinoma	Cells	Promote death (60)
	TGR5-CREB-cyclin D1	Endometrial cancer	Cells	Promote proliferation (61)
	FXR	Cholangiocarcinoma	Cells and mice	Suppress proliferation (62)
UDCA	Intrinsic mitochondrial apoptotic cell death pathway	Melanoma	Cells	Promote death (63)
	Autophagy	Gastric carcinoma	Cells	Promote death (64)
	ER stress	Glioblastoma multiforme	Cells	Promote death (65)
	DLC1	Hepatocellular carcinoma	Cells	Suppress proliferation (66)
	Intrinsic mitochondrial apoptotic cell death pathway/extrinsic death receptor pathway of apoptosis	Prostate Cancer	Cells	Promote death (67)
	Oxidative stress	Colorectal cancer	Cells	Suppress proliferation (68)
Conjugated bile acids (CBAs)	NF-kB	Cholangiocarcinoma	Cells and mice	Promote proliferation (69)
Free bile acids	NF-kB	Cholangiocarcinoma	Cells and mice	Suppress proliferation and promote death (69)

## Bile Acids in Cancer Cell Invasion and Metastasis

Invasion and metastasis are major events and typical features in the later stages of cancer progression, which seriously threaten the lives of cancer patients (70). In fact, the properties of invasion and metastasis are the primary hallmarks that distinguish benign tumors from malignant tumors, some exceptions notwithstanding (71, 72). Cancer invasion is regarded as the first critical step of metastasis, which refers to the detachment of tumor cells from the primary location, followed by invasion and destruction of adjacent normal tissues (73, 74). This process occasions several principal molecular events, involving changes in cell-cell and cell-extracellular matrix (ECM) adhesion, the release of proteolytic enzymes and the dissolution of ECM, and the motility of tumor cells to move through the tissue (73, 75). Cancer metastasis is a series of non-linear, parallel, and partially overlapping processes, defined as the spread and colonization of cancer cells from the primary neoplasm to distant tissues or organs (76). Cancer metastasis is certainly 1) initiated by cancer cells invading the tissue surrounding the primary neoplasm, 2) subsequently intravasate into tumor vasculature, 3) evade surveillance and survive during vascular transport, 4) being arrested by distant organs and extravasate into the parenchyma of tissue, 5) survival to form micrometastases, 6) initiate proliferation signals to generate macrometastases that can be monitored clinically (77, 78).

The performance of bile acids in cancer invasion and metastasis has become increasingly prominent, although the role seems to be ambiguous (**Table 3**). In this regard, bile acids are more commonly reported for their pro-invasion and pro-metastasis effects. Specifically, Baek et al. found that LCA enhances the invasiveness of human colon cancer cells by up-regulating uPAR expression (79). Halvorsen et al. also obtained similar results, that LCA promoted the secretion of matrix metalloproteinase 2 (MMP-2) and increased cellular invasion in human colon cancer cells (80). For DCA, several studies have confirmed its ability to promote invasion/migration in colon cancer and esophageal adenocarcinoma, but only at a low concentration (20 μM) (82, 83). Pai et al. concluded that at doses of 5 and 50 μM, DCA can enhance the invasiveness of colon cancer cells by activating β-catenin–cyclin D1 and –uPAR signaling pathways (55). Unlike the direct effect on cancer cells, Nguyen et al. found that DCA initially induced hepatic stellate cells to secrete senescence-associated secretory phenotype (SASP) factors, thereby indirectly promoting the invasion/migration of liver cancer cells (11). Regarding CDCA, Wu et al. described that it mediates the enhancement of gastric carcinoma cell invasion by activating PKC and COX-2 signals (86). And Debruyne et al. found that all LCA, DCA, and CDCA other than UDCA can stimulate the invasiveness of colorectal cancer cells by activating SRC oncogene and Rho-like small GTPases (81). In contrast, Liu et al. reported that more hydrophilic CBAs, rather than free bile acids, promote the invasive growth of cholangiocarcinoma (CCA) cells (90). When it comes to the anti-invasive/metastatic potential of bile acids, UDCA bears the brunt due to its therapeutic properties (although reports are limited), but the role of other bile acids cannot be ruled out. For example, Quilty et al. concisely described the phenomenon that high-dose (200 μM) DCA inhibits the invasiveness of esophageal adenocarcinoma in a study (83). Pyo et al. proposed for the first time that a physiological concentration (100 μM) of DCA can frustrate the invasion and migration of gastric carcinoma cells through the inhibition of Snail and MMP9, as well as the induction of E-cadherin and MUC2 (84). And Phelan et al. also provided evidence of the anti-invasion/migration of DCA and CDCA in prostate cancer (85). For UDCA, Wu et al. found that treatment with UDCA can attenuate the invasiveness of gastric carcinoma cells induced by CDCA by interfering with the generation of PGE2 (86). Kim et al. also verified the anti-metastatic effect of UDCA in pancreatic cancer, which is executed by inhibiting the levels of intracellular ROS and Prx2 and reducing the epithelial-mesenchymal transition (EMT) of pancreatic cancer cells (87). In addition, tauroursodeoxycholic acid (TUDCA), a conjugated form of UDCA, manifests anti-invasive impacts related to the decreased expression of MMP-7 and -13 in metastatic breast cancer, whether in normoxia or hypoxia (88). Paradoxically, Jia et al. found that the concentration of CBAs, including TUDCA, was higher in patients with intrahepatic cholangiocarcinoma with vascular invasion, compared to patients with intrahepatic cholangiocarcinoma without vascular invasion, while free bile acids were observed the opposite result (89). According to the above evidence, the concentration and type of BAs and cancer classification are still the dominant factors defining the distinct fates of cancer. For instance, LCA and DCA habitually promote cancer invasion and metastasis within a concentration of 30 μM, while DCA exerts an inhibitory impact at >100 μM. For CDCA, the executive dose that promotes the invasiveness of gastric cancer cells is 200 μM, and the maximum dose that stimulates the invasion of colorectal cancer cells is 10 μM. Regardless, the role of bile acids in cancer cell invasion/metastasis is antagonistic and interesting.

**Table 3:** The impact of bile acid on cancer cell Invasion and metastasis.

Bile acid	Target	Cancer	Model(s)	Effect
LCA	Erk-1/2, AP-1-uPAR	Colorectal cancer	Cells	Promote (79)
	Matrix metalloproteinase	Colorectal cancer	Cells	Promote (80)
	SRC, Rho-like small GTPases	Colorectal cancer	Cells	Promote (81)
DCA	SRC、Rho-like small GTPases	Colorectal cancer	Cells	Promote (81)
	Protein kinase C	Colorectal cancer	Cells	Promote (82)
	β-catenin–cyclin D1 and –uPAR	Colorectal cancer	Cells	Promote (55)
	COX-2	Colorectal cancer	Cells	Promote (57)
	Matrix metalloproteinase	esophageal Adenocarcinoma	Cells	Promote (83)
	Hepatic stellate cells (HSCs)	Hepatocellular carcinoma	Cells and humans	Promote (11)
	/	esophageal Adenocarcinoma	Cells	Suppress (83)
	Snail, MMP9, E-cadherin and MUC2	Gastric carcinoma	Cells and humans	Suppress (84)
	HIF-1α	Prostate cancer	Cells	Suppress (85)
CDCA	PKC-COX-2	Gastric carcinoma	Cells	Promote (86)
	SRC, Rho-like small GTPases	Colorectal cancer	Cells	Promote (81)
	HIF-1α	Prostate cancer	Cells	Suppress (85)
UDCA	PGE2	Gastric carcinoma	Cells	Suppress (86)
	ROS, Prx2 and STAT3	Pancreatic cancer	Cells	Suppress (87)
TUDCA	MMP-7 and MMP-13	Breast cancer	Cells	Suppress (88)
Free bile acids	/	Intrahepatic cholangiocarcinoma	Humans	Suppress (89)
CBAs	/	Intrahepatic cholangiocarcinoma	Humans	Promote (89)
	S1PR2-ERK1/2	Cholangiocarcinoma	Cells and organoids	Promote (90)

## Bile Acids in Cancer Inflammation and Immunity

Inflammation, especially chronic inflammation, has long been associated with cancer (91, 92). Inflammation is the body’s gradual formation of immune defense response to resist foreign pathogens and cope with tissue damage in the long evolutionary process and is mainly characterized by the vascular response, the recruitment of immune cells, and the release of cytokines (93). In fact, tumors can be regarded as unhealable wounds in a certain sense (94). As one of the characteristics of cancer, inflammation is conducive to acquiring core hallmarks of cancer, including immortal proliferation signals, angiogenesis, invasion, metastasis, etc (3). Inflammation implements these cancer hallmark-facilitating programs through the tumor microenvironment (TME). TME, the internal environment for tumor cells to establish and survive, contains a roster of components including fibroblasts, immune and inflammatory cells (such as macrophages, T and B lymphocytes, etc.), endothelial cells, and other cells, as well as microvessels and biomolecules infiltrating them (3, 95). All these cells can deliver an assorted array of cytokines to maintain the inflammatory environment for cancer cell survival and weaken the anti-tumor immune response (96). Generally, the immune system is executed to eradicate damaged cells and combat foreign pathogens. Yet, it is fascinating that the immune cells in TME also seem to be involved in the tumor-promoting process through a complicated regulatory network (95).

Bile acids have long been classified as tissue damage and pro-inflammatory molecules due to their capacity to stimulate the secretion of a variety of cytokines and chemokines (97). More and more emerging studies have shown that bile acids dysregulation is involved in the regulation of inflammation and immunity (**Table 4**). Cancer-related inflammation is related to carcinogenesis through so-called bridging factors, including signaling pathways NFκB, COX-2, STAT3, and so on. For example, CA-treated mice developed low-grade enteritis (indicated by the overexpression of inflammatory factors such as IL-6, IL-1β, and TNF-α) and activation of STAT3 signaling, which promoted subsequent intestinal carcinogenesis (99). Similarly, TCA, a conjugated form of CA, was perceived to be positively correlated with the level of the inflammatory factor IL-4 in intrahepatic cholangiocarcinoma (89). Also, exposure to DCA and CDCA can up-regulate the expression of pro-inflammatory genes, including COX2, related to tumorigenesis and development (108). In contrast, CDCA was shown to down-regulate the expression of IL-6 and COX2 in cholangiocarcinoma, while GDCA (a conjugated form of DCA) is the opposite (69). In addition to acting directly on cancer cells, primary bile acids instead of secondary bile acids can also control the recruitment of natural killer T (NKT) cells in the liver by promoting the expression of chemokine CXCL16 and thus exerting anti-tumor immune functions to inhibit the growth of liver cancer (100). Similarly, in breast cancer, LCA feeding enhances the anti-tumor immune response by increasing the number of tumor-infiltrating lymphocytes (TILs) in mice (45). Several studies have also found that DCA initially induces hepatic stellate cells (HSCs) to fabricate senescence-associated secreted phenotype (SASP) factors, which in turn stimulates the secretion of pro-inflammatory and tumor-promoting factors, and is ultimately responsible for the development of non-alcoholic steatohepatitis (NASH) and subsequent liver cancer (11, 98). Furthermore, the performance of bile acid-activated receptors (BARs) and transporters in inflammation and immune regulation has also been extensively reported. The most characteristic members of BARs are specific nuclear receptors (the most representative of FXR) and G protein-coupled receptors (GPBAR1 is the most familiar, also known as TGR5) (109). Studies have found that the attenuation of FXR signals can induce liver bile acids retention and persistent inflammation by down-regulating the function of bile acid transporters, thereby promoting the development of liver cancer (27, 102–105). Conversely, treatment of non-alcoholic steatohepatitis-hepatocellular carcinoma (NASH-HCC) mice with cholestyramine, a bile acid sequestrant, can significantly inhibit the development of liver cancer by promoting the excretion of hydrophobic bile acids (101). And bile acid-TGR5 signal axis can balance the generation of pro-inflammatory and anti-inflammatory cytokines by regulating the polarization state of macrophages, consequently controlling subsequent gastrointestinal carcinogenesis (27, 106, 107). Succinctly, BAs stimulate/decrease the secretion of inflammatory factors such as IL-6 and TNF-α on the one hand, thereby activating/inactivating signal pathways related to cancer promotion to improve/inhibit cancer growth or invasiveness. On the other hand, BAs foster a tumor-rejecting environment by regulating the recruitment of immune cells such as NKT and TILs or the polarization state of macrophages, thereby controlling the proliferation and invasion of cancer. Although the role of bile acids in inflammatory immune regulation is not uncommon, the specific regulatory mechanism needs to be further explored.

**Table 4:** The impact of bile acid on cancer inflammation and immunity.

Bile acid	Target	Cancer	Model(s)	Effect
LCA	Tumor-infiltrating lymphocytes (TILs)	Breast cancer	Mice	Suppress (45)
DCA	Hepatic stellate cells (HSCs)	Hepatocellular carcinoma	Cells and humans	Promote (11, 98)
CA	IL-6, IL-1β, TNF-α and STAT3	Colorectal cancer	Cells and mice	Promote (99)
TCA	IL-4	Intrahepatic cholangiocarcinoma	Humans	Promote (89)
CDCA	IL-6 and COX2	Cholangiocarcinoma	Cells and mice	Suppress (69)
GDCA	IL-6 and COX2	Cholangiocarcinoma	Cells and mice	Promote (69)
Primary BAs	Natural killer T (NKT) cells	Hepatocellular carcinoma	Mice and humans	Suppress (100)
Hydrophobic BAs	FXR, BSEP and CYP7A1	Hepatocellular carcinoma	Cells and mice	Promote (101)
TBAs	FXR, bile acid transporters	Hepatocellular carcinoma	Mice and humans	Promote (27, 102–105)
	TGR5, macrophages	Colorectal cancer	Cells, mice, and humans	Promote/suppress (27, 106, 107)

## Conclusions

Are bile acids foes or friends? This has been a controversial subject for a long time. Our knowledge of bile acids has been extended from promoting the absorption of lipids to key signaling molecules that maintain the body’s homeostasis, albeit in its infancy. Especially in the field of cancer, bile acids are bestowing more and more surprises in angiogenesis, cancer cell proliferation and death, tumor invasion and metastasis, inflammation and immune regulation, etc. Based on the special amphipathic nature and the wide variety of categories, bile acids play opposite roles in separate cancers, even the same cancer. This contradictory role endows bile acids with a mysterious content. The cytotoxic properties of bile acids enable us to further explore to develop potential drugs for cancer treatment. At present, UDCA has the greatest prospects as a drug, not only can directly exert its anti-tumor effect but also can inhibit the development of inflammation-tumor sequence by reducing the proportion of toxic bile acids (such as DCA) (110, 111). The amphiphilic nature of bile acid allows us to apply it as a medium for coupling with other chemopreventive agents to improve drug absorption. Of course, bile acids and BARs can also be handled as therapeutic targets, although it is not straightforward to formulate a strategy to act on one receptor in a specific cell type. Simply put, the paradoxical role of bile acids in cancer gives us unlimited possibilities for exploration. Consequently, this review aims to encourage the emergence of more intensive studies on the regulation of bile acids in tumor progression.

## Author Contributions

All authors listed have made a substantial, direct, and intellectual contribution to the work, and approved it for publication.

## Funding

This work was supported by the Major Projects of Jinhua Science and Technology Plan Project (No. 2018-3-001a), Special Research Fund for Basic Research of Jinhua Central Hospital (JY2020-6-11), and Social Development Project of Public Welfare Fund Research of Zhejiang Province (2015C33253).

## Conflict of Interest

The authors declare that the research was conducted in the absence of any commercial or financial relationships that could be construed as a potential conflict of interest.

## Publisher’s Note

All claims expressed in this article are solely those of the authors and do not necessarily represent those of their affiliated organizations, or those of the publisher, the editors and the reviewers. Any product that may be evaluated in this article, or claim that may be made by its manufacturer, is not guaranteed or endorsed by the publisher.
